# The Effect of Liraglutide on Weight Loss in Women with Polycystic Ovary Syndrome: An Observational Study

**DOI:** 10.3389/fendo.2014.00140

**Published:** 2014-08-27

**Authors:** Christina B. Rasmussen, Svend Lindenberg

**Affiliations:** ^1^Copenhagen Fertility Center, Copenhagen, Denmark

**Keywords:** polycystic ovary syndrome, weight loss, obesity, liraglutide, GLP-1 analogs

## Abstract

**Objective:** The aim of the present study was to evaluate the effect of the glucagon-like peptide-1 analog liraglutide on weight loss in overweight and obese women with polycystic ovary syndrome (PCOS).

**Methods:** In an observational study, 84 overweight or obese women with PCOS were treated with liraglutide. Baseline characteristics and weight changes at clinical follow-up were recorded. Main outcome measures were absolute and relative weight loss.

**Results:** In overweight or obese women with PCOS treated with liraglutide for a minimum of 4 weeks, a mean weight loss of 9.0 kg (95% CI: 7.8–10.1, *p* < 0.0001) and a mean decrease in BMI of 3.2 kg/m^2^ (95% CI: 2.8–3.6, *p* < 0.0001) were found. A weight loss of more than 5 and 10% of baseline weight was seen in 81.7 and 32.9% of patients, respectively. The mean duration of treatment with liraglutide was 27.8 weeks (SD 19.2).

**Conclusion:** Treatment with liraglutide in combination with metformin and lifestyle intervention resulted in a significant weight loss in overweight and obese women with PCOS, indicating that liraglutide may be an effective alternative for weight loss in this group of patients. However, larger placebo-controlled studies are needed to confirm this.

## Introduction

Polycystic ovary syndrome (PCOS) is the most common endocrine disorder in women of reproductive age, with a reported prevalence of 6–15% depending on the population studied and the diagnostic criteria used (1–3). According to the Rotterdam criteria introduced in 2003, PCOS is defined by at least two of the following: oligo-amenorrhea, clinical or biochemical hyperandrogenism and polycystic ovaries on ultrasound (4). A large proportion of women with PCOS are overweight or obese, in particular with abdominal obesity (5). They often have insulin resistance, compensatory hyperinsulinemia, impaired glucose tolerance, and a higher risk of type 2 diabetes (6–8). PCOS is therefore considered as a prediabetic state. Although PCOS and obesity are strongly related, the underlying mechanism linking the two involves a complex pathophysiology. Obesity may play a pathogenic role in the development of PCOS in susceptible individuals. Obesity-related insulin resistance and resulting hyperinsulinemia may cause a decreased sex-hormone binding globulin production and an increased ovarian androgen production, both of which contribute to the hyperandrogenism. However, this may form a vicious circle as hyperandrogenism may also contribute to the insulin resistance by increasing free fatty acid flux to the liver and muscle through visceral lipolysis and, in addition, by altering muscle structure toward less insulin-sensitive muscle fibers (5). In addition, PCOS is associated with dyslipidemia and endothelial dysfunction (9, 10). The most pronounced metabolic abnormalities are seen in women with hyperandrogenism and obesity, especially central obesity (8, 11).

Even a modest weight loss in overweight or obese women with PCOS reduces insulin resistance, hyperinsulinemia, and hyperandrogenism and increases sex-hormone binding globulin production (12–14), thereby improving hirsutism, menstrual cyclicity, ovulation rates, and fertility. Especially, loss of intra-abdominal fat is associated with resumption of ovulation (15). Furthermore, weight loss has beneficial effects on cardiovascular risk factors such as dyslipidemia and blood pressure (12). Therefore, weight reduction is essential in overweight and obese women with PCOS.

Liraglutide, a glucagon-like peptide-1 (GLP-1) analog with 97% structural homology to human GLP-1 is used in the treatment of type 2 diabetes. GLP-1 is a gut hormone of the incretin family that enhances glucose-stimulated insulin secretion, inhibits glucagon secretion, delays gastric emptying, and reduces food intake and appetite. In type 2 diabetes, liraglutide significantly lowers HbA1c and, in addition, reduces body weight (16). A recent meta-analysis concluded that GLP-1 receptor agonists not only had a significant effect on weight loss in overweight type 2 diabetic patients but also in non-diabetic overweight persons (17). Only one smaller study has investigated the effect of liraglutide in PCOS patients reporting a significantly greater weight loss with liraglutide in combination with metformin than metformin alone (18). Furthermore, a beneficial effect on blood pressure and cholesterol levels has been observed with GLP-1 analogs (16, 17).

Given that PCOS is a frequent condition and weight loss is essential but difficult to achieve, it is important to study if the effect on body weight reported in other studies can be confirmed in a selected population of PCOS patients, especially since liraglutide is not currently approved for weight reduction. In this study, we therefore investigated the effect of liraglutide on weight reduction in a larger cohort of PCOS patients.

## Materials and Methods

The study population comprises all overweight or obese women starting treatment with liraglutide in the period August 2010 to January 2012 in a private gynecology and fertility clinic. The following variables were recorded: date of first prescription, age, body weight at start of treatment, height, weight loss at clinical follow-up, and dose of liraglutide. Patients were all diagnosed with PCOS according to the Rotterdam criteria, were overweight or obese, and had failed to lose any weight after 6 months with metformin and life style intervention. The intervention consisted of a low-glycemic diet with no caloric restriction, guidance by a dietitian, and a recommendation of 45 min of moderate exercise for a minimum of three times per week. Treatment with metformin was given due to insulin resistance and was therefore continued after start of liraglutide. The procedures of the study were in accordance with the Helsinki Declaration, and all patients were informed of the off-label use of liraglutide and gave written informed consent.

Duration of treatment was calculated in full weeks from date of prescription to discontinuation of treatment or date of last registered weight loss for patients still being treated. Body weight and weight loss were self-reported. In patients who temporarily stopped treatment and gained weight in the pause, weight loss was calculated as net weight loss over the entire period. Starting dose was 0.6 mg given as a subcutaneous injection once daily. Dose was increased if no effect on weight (i.e., weight loss 0 kg) and no side effects, initially to 1.2 mg and later to 1.8 mg.

Descriptive characteristics are reported as mean and standard deviation (SD). Mean value with corresponding 95% confidence interval for changes in body weight and BMI was estimated using Student’s paired *t*-test. Level of statistical significance was set at *p* < 0.05 and all *p*-values were two-sided. Data analysis was conducted in SAS, version 9.1 (SAS Institute, Cary, NC, USA).

## Results

**Figure 1:**
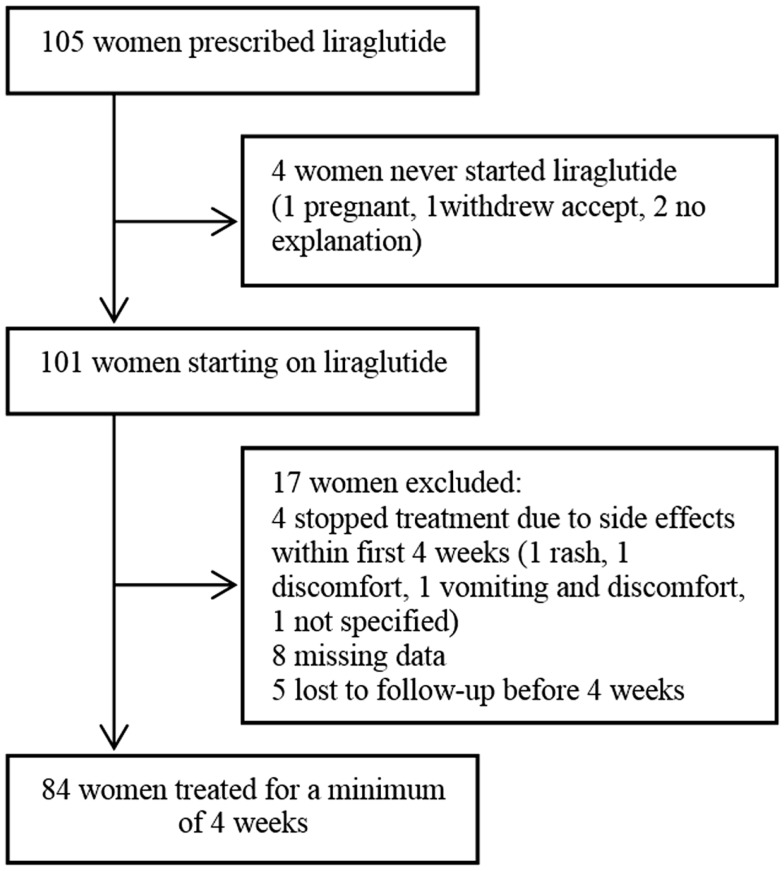
**Overview of women included in the study**.

A total of 105 overweight or obese women with PCOS had a prescription of liraglutide. We excluded four patients who never used the prescription. Of the 101 patients starting on liraglutide, four patients (4.0%) were excluded because of discontinuation of treatment due to side effects after less than 4 weeks, eight patients (7.9%) were excluded because of missing data, and finally five patients (5.0%) were lost to follow-up after <4 weeks and were excluded, leaving 84 patients for analysis (Figure 1).

Mean age at start of treatment was 35.5 years (SD 7.9). The patients had a mean body weight at baseline of 98.9 kg (SD 17.0) (Table 1). For two patients, no data on baseline weight could be obtained, thus BMI could not be calculated. Of the remaining 82 women, 67 (81.7%) were obese defined as BMI ≥ 30 and 15 (18.3%) were overweight with BMI between 25 and 30. Mean BMI at baseline was 35.0 kg/m^2^ (SD 6.0). Mean duration of treatment was 27.8 weeks (SD 19.2). Twenty women (23.8%) were trying to lose weight prior to fertility treatment and six women (7.1%) had previously been treated for infertility. Two patients (2.4%) were diagnosed with diabetes.

**Table 1:** **Changes in body weight and BMI after treatment with liraglutide in overweight and obese women with PCOS**.

	Baseline	After treatment with liraglutide	Decrease	95% CI	*p*
Body weight (kg)	98.9 (17.0)	89.8 (17.8)	9.0 (5.3)	7.8–10.1	<0.0001
BMI (kg/m^2^)	35.0 (6.0)	31.8 (6.2)	3.2 (1.9)	2.8–3.6	<0.0001

*Values are mean (SD). Student’s paired *t*-test was used to estimate mean difference from baseline with corresponding 95% confidence intervals (CI) and *p*-values*.

*BMI, body mass index*.

Overall, we observed a significant weight loss in women with PCOS after treatment with liraglutide, the mean weight loss being 9.0 kg (95% CI: 7.8–10.1, *p* < 0.0001). Likewise, a significant mean decrease in BMI of 3.2 kg/m^2^ (95% CI: 2.8–3.6, *p* < 0.0001) was found (Table 1). Two patients lost no weight, but no patients experienced a net weight gain. In all, 67 women (81.7%) lost more than 5% and 27 women (32.9%) lost more than 10% of their baseline weight. The mean relative weight loss was 9.4% (95% CI: 8.2–10.6, *p* < 0.0001) of baseline weight.

A dose of 1.8 mg was reached at some point during treatment in 52/84 (61.9%) patients. As interval to increase in dose differed substantially among patients and some patients had multiple increases and decreases, no comparisons between dose groups have been made.

**Figure 2:**
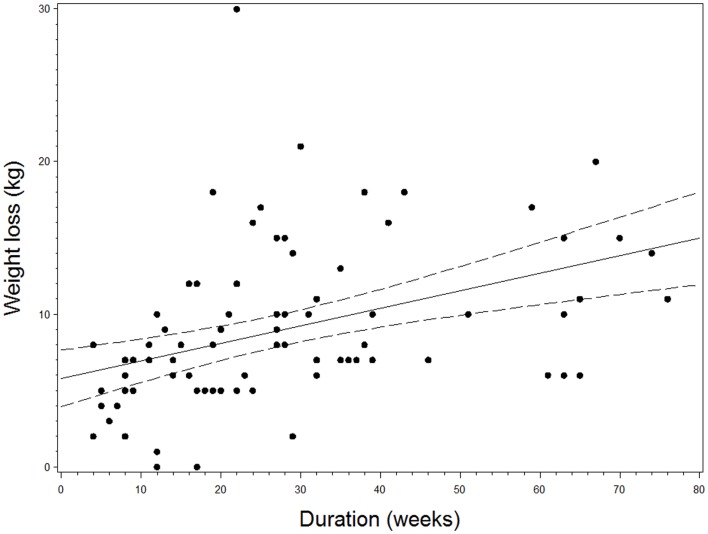
**Scatterplot of the relation between duration of treatment with liraglutide and weight loss with best fitted right line and 95% confidence interval**.

A total of 50/84 patients (59.5%) were treated for a minimum of 20 weeks. When restricting analysis to patients treated for 20 weeks or more, mean weight loss was 10.9 kg (95% CI: 9.4–12.5, *p* < 0.0001) and mean decrease in BMI was 3.9 kg/m^2^ (95% CI: 3.3–4.4, *p* < 0.0001) after a mean duration of 39.1 weeks (SD 17.0). Figure 2 shows weight loss in relation to duration of treatment.

Treatment with liraglutide was terminated for the following reasons: achieving the desired weight loss in 10 women (11.9%), side effects in four women (4.8%), start of fertility treatment or pregnancy in five women (6.0%), no further effect of liraglutide in 11 women (13.1%), moving or traveling in two women (2.4%), and in one woman (1.2%) no reason was given. Six women (7.1%) were lost to follow after more than 4 weeks of treatment. These women were included in the analysis with data from their last clinical visit. The remaining 45 women (53.6%) were still being treated with liraglutide when conducting the present study.

A total of 19 women (22.6%) reported side effects. Nausea was reported by six (7.1%), vomiting by one (1.2%), diarrhea by four (4.8%), constipation by one (1.2%), abdominal pain/discomfort by six (7.1%), itching by two (2.4%), and discomfort by four (4.8%). In another four women (4.8%), side effect was not specified. Dose was reduced in nine women (10.7%) due to side effects.

## Discussion

The prevalence of obesity is increasing worldwide. Due to its weight reducing effect, the potential role of liraglutide in the treatment of obesity is currently being debated including its role in PCOS-related obesity (19, 20). In the present study, treatment with liraglutide in 84 overweight and obese women with PCOS resulted in a significant weight loss of 9.0 kg. Only one smaller study has evaluated the effect of liraglutide in PCOS and, in agreement with our findings, showed a beneficial effect on body weight (18). In a 12-week open-label study of 36 obese women with PCOS, mean weight losses of 6.5, 3.8, and 1.2 kg with liraglutide 1.2 mg plus metformin, liraglutide 1.2 mg alone, and metformin alone, respectively, were reported. Weight loss with combination therapy was significantly greater than with metformin alone. However, there were only 11 patients in each of the two groups treated with liraglutide. A meta-analysis of GLP-1 receptor agonists in type 2 diabetes and obesity, including eight trials using liraglutide, also demonstrated a positive effect on weight loss (17). In type 2 diabetes, mean weight losses of 2.05, 2.45, and 2.8 kg after 52 weeks of liraglutide 1.2, 1.8, and 26 weeks of liraglutide 1.8 mg in combination with metformin, respectively, have been reported (16, 21, 22). In obese non-diabetic subjects, liraglutide in doses of 1.2, 1.8, 2.4, and 3.0 mg resulted in mean weight losses of 4.8, 5.5, 6.3, and 7.2 kg, respectively, after 20 weeks (23).

In our study, 81.7% of patients lost more than 5% of baseline weight. In comparison, a 20-week randomized study of liraglutide in obese non-diabetic subjects demonstrated a relative weight loss of 5% or more in 61% of individuals treated with liraglutide in doses of 1.2–3.0 mg, with a higher proportion achieving this in the highest dose group (23). In an extension of that trial, 64% of subjects on liraglutide lost >5% of body weight after 1 year and 85% of these maintained that weight loss after 2 years of treatment (24).

Differences in the magnitude of the effect of liraglutide on weight loss between studies may be due to differences in study populations and design. The population in our study is not comparable to most trials on liraglutide, which have been done on a more diverse population with greater mean age and also including men. Although, similar weight losses with liraglutide have been observed in men and women (24), and none of the studies have reported weight loss in relation to age. All women in our study were taking metformin simultaneously. Slightly, higher mean weight reductions have been reported in trials of liraglutide in combination with metformin than trials of liraglutide alone (21, 22), but no larger studies with direct comparison of liraglutide monotherapy versus combination therapy with metformin could be identified. Except for metformin, most antidiabetic drugs are associated with weight gain potentially augmenting the effect of liraglutide in studies in diabetic populations. Only two patients in our study had diabetes. Furthermore, some women in the present study were referred for infertility and could be more motivated to make favorable changes in lifestyle in order to improve chance of pregnancy and possibly also be willing to accept side effects to a greater extent compared to type 2 diabetics or obese non-diabetics. Additionally, we excluded patients dropping out before four full weeks of treatment, whereas most of the above mentioned trials have been based on analyses of the intention-to-treat population. Finally, weight loss on liraglutide is dose-dependent with greater loss with increasing dose (23, 25), and 61.9% of patients in the present study received a dose of 1.8 mg.

Our results are also in agreement with a study evaluating the effect of another GLP-1 receptor agonist in PCOS. A 24-week randomized study of exenatide demonstrated a mean weight loss of 3.2, 6.0, and 1.6 kg with monotherapy, combination therapy with metformin, and metformin alone, respectively, and in both exenatide arms was the difference from metformin alone statistically significant (26). Liraglutide has been shown to decrease HbA1c more than exenatide, but no difference in weight loss was observed (25). However, the exenatide study had only 20 patients in each of the 3 arms and thus had limitations of small sample size and also a high drop-out rate of 30%.

A large proportion of women with PCOS are overweight or obese and infertility due to anovulation is common. Significant improvements in insulin resistance, hyperinsulinemia, dyslipidememia, menstrual cyclicity, ovulation rates, and fertility have been shown with a modest relative weight loss of 5–10% in PCOS (12–15, 27, 28). Therefore, weight reduction is essential in the management of PCOS. As 81.7 and 32.9% of patients in the present study lost more than 5 and 10% of baseline weight, respectively, it is possible that improvements in these parameters have occurred. However, we had no information on metabolic and reproductive changes, and were thus unable to investigate any potential effect of weight loss in this aspect. Exenatide combined with metformin in PCOS significantly improved ovulation rates and metabolic parameters (decreased free androgen index, total cholesterol and trigyceride, increased insulin sensitivity measures) and reduced abdominal girth compared with metformin alone (26). In obese non-diabetic subjects, liraglutide has been shown to reduce the prevalence of prediabetes with 84–96% and the proportion of patients with metabolic syndrome by more than 60% (23). Although no significant effect on menstrual frequency and most metabolic and hormonal parameters were observed in women with PCOS using liraglutide for 12 weeks, this is possibly due to the short duration of that study (18).

Gastrointestinal side effects such as diarrhea, vomiting and, in particular, nausea are common with liraglutide treatment. Nausea has been reported by 11–48% and vomiting by 4–15% of subjects taking liraglutide, and like the weight reduction it occurs in a dose-dependent manner (21–25). It could therefore be speculated whether the weight reducing effect of liraglutide could be ascribed to these side effects. However, nausea mostly occurred in the first 4–6 weeks of treatment, was transient, and of mild to moderate intensity (21–25). In the present study, frequency of side effects was generally low when compared to other studies of liraglutide. Reasons for this could include a slower dose titration, but also our exclusion of patients who did not complete 4 weeks of treatment, among these four women with side effects. Additionally, some women lost to follow-up could have dropped out because of side effects. Garber et al. found no difference in weight loss between patients with and without persistent nausea (defined as ≥7 days) with liraglutide 1.2 and 1.8 mg (21). Similar results were found in a *post hoc* analysis of the study by Astrup et al. where the difference in weight loss between individuals with and without nausea or vomiting was significant only for the group on liraglutide 3.0 mg (29). Furthermore, the dose-dependent weight reduction with increasing doses of liraglutide was maintained both for participants with and without nausea or vomiting. Finally, weight loss among patients without nausea or vomiting was still significantly greater with liraglutide than with placebo or orlistat (24). Thus, nausea may increase weight loss some in high doses of liraglutide, but does not seem to be a major contributor for the weight reduction seen with liraglutide.

All patients in our study were taking metformin simultaneously. Gastrointestinal side effects are also frequent with metformin. It is therefore noteworthy that studies investigating combination therapy of liraglutide and metformin did not report higher frequencies of nausea or vomiting than studies of liraglutide monotherapy (21, 22).

A limitation of our study is the lack of a placebo group. Secondly, weight loss was self-reported, potentially reducing the accuracy of the effect. Furthermore, weight loss from our study is difficult to compare with studies of a fixed duration because of great variations in duration of treatment among women in our study. Treatment duration ranged from 4 to 76 weeks and weight loss tended to increase with increasing duration of treatment with liraglutide, probably contributing to the differences in weight loss between subjects. Most studies have evaluated effect after at least 20 weeks of treatment. We tried to compensate for this by conducting a subgroup analysis restricting duration to 20 weeks or more, resulting in a slightly higher mean weight loss. In addition, all women had initially been treated with metformin and lifestyle intervention with a low-glycemic diet and exercise for 6 months, and only women who did not lose any weight on this regimen were prescribed liraglutide. After starting liraglutide, women were encouraged to continue the lifestyle intervention. We cannot rule out that this has contributed to the weight loss or that adherence to lifestyle intervention might have changed. However, we do not believe this to be the primary explanation for the weight loss. Finally, the lack of information on improvements in reproductive and metabolic dysfunction limits assessment of the clinical effect of weight loss. This should be addressed in future studies.

In summary, the results of this study indicate that liraglutide in combination with metformin and lifestyle intervention may be an effective alternative for weight loss in women with PCOS who fail to lose weight on diet, exercise, and metformin. However, larger prospective placebo-controlled intervention studies in overweight and obese women with PCOS are needed to establish the effect on weight loss. The clinical effect of weight loss on reproductive and metabolic parameters should be investigated. Finally, studies on sustainability of the achieved weight loss are needed.

## Conflict of Interest Statement

The authors declare that the research was conducted in the absence of any commercial or financial relationships that could be construed as a potential conflict of interest.

## Abbreviations

BMI, body mass index; CI, confidence interval; GLP-1, glucagon-like peptide-1; PCOS, polycystic ovary syndrome; SD, standard deviation.
